# Experiences of parenting and clinical intervention for mothers affected by personality disorder: a pilot qualitative study combining parent and clinician perspectives

**DOI:** 10.1186/s12888-018-1733-8

**Published:** 2018-05-25

**Authors:** Ruth Wilson, Tim Weaver, Daniel Michelson, Crispin Day

**Affiliations:** 1 Community Eating Disorder Service, East London NHS Foundation Trust, London, UK; 2 Department of Mental Health, Social Work and Integrative Medicine, Middlesex University, London, UK; 3 Department of Population Health, Centre for Global Mental Health, London School of Hygiene & Tropical Medicine, London, UK; 4 CAMHS Research Unit, IOPPN, King’s College London, Michael Rutter Centre, De Crespigny Park, Camberwell, London, SE5 8AZ UK

**Keywords:** Parenting, Personality disorder, Child behaviour, Child emotional problems

## Abstract

**Background:**

Evidence-based parenting programmes are recommended for the treatment of child mental health difficulties. Families with complex psychosocial needs show poorer retention and outcomes when participating in standard parenting programmes. The *Helping Families Programme* (HFP) is a 16-week community-based parenting intervention designed to meet the needs of these families, including families with parental personality disorder. This study aimed to explore the help seeking and participatory experiences of parents with a diagnosis of personality disorder. It further aimed to examine the acceptability of referral and intervention processes for the HFP from the perspectives of (i) clinicians referring into the programme; and (ii) referred parents.

**Method:**

Semi-structured interviews were conducted with parents recruited to receive HFP (*n* = 5) as part of a research case series and the referring NHS child and adolescent mental health service (CAMHS) clinicians (*n* = 5). Transcripts were analysed using Interpretive Phenomenological Analysis.

**Results:**

Four themes were identified for parents: (i) the experience of parenthood, (ii) being a parent affected by personality disorder, (iii) experience of the intervention, and (iv) qualities of helping. Three themes emerged for clinicians: (i) challenges of addressing parental need, (ii) experience of engaging parents with personality disorders and (iii) limited involvement during HFP. Comparison of parent and clinician themes led to the identification of two key interlinked themes: (i) concerns prior to receiving the intervention, and (ii) the challenges of working together without a mutual understanding.

**Conclusions:**

This pilot study identifies potentially significant challenges of working with parents affected by personality disorder and engaging them in HFP and other similar interventions. Results have important wider clinical implications by highlighting potential barriers to engagement and participation and providing insights on how these barriers might be overcome. Findings have been used to inform the referral and intervention processes of a pilot RCT and further intervention development.

## Background

One in ten children in developed economies experience emotional, behavioural and other mental health disorders. These disorders have negative impacts on childhood development, academic achievement and social functioning and are associated with subsequent adult mental health difficulties, long-term unemployment and criminal behaviour [1–3]. Parental personality disorder increases the likelihood of child mental health problems and maltreatment. Associated parental problems include increased emotional dysregulation, hostile interpersonal functioning, self-harm and substance misuse [4–6]. Potentially harmful parenting behaviours such as excessive parental control, possessiveness and physical punishment have also been documented [7] that can interfere with child-parent attachment and undermine parents’ capacity to provide children with warm, nurturant and consistent parenting.

Families with complex psychosocial needs are likely to experience poorer outcomes from established parenting programmes based on social learning theory [8–11]. Parental mental health has also been shown to moderate the effectiveness of parenting interventions with families showing less improvement in child problem behaviours at the end of treatment [10, 12]. Support for families with complex psychosocial needs is often fragmented, not tailored and personalised, and fails to address the interplay between parenting and specific parental emotional and interpersonal functioning [13–15].

The *Helping Families Programme* (HFP) is a modular parenting programme for parents with complex psychosocial needs, including personality disorder [16, 17]. HFP aims to improve i) child mental health and behavioural problems, ii) parent-child relationships, iii) parental emotion regulation and coping and iv) families’ social resources. HFP incorporates validated therapeutic content focussed on parenting, emotional regulation and interpersonal functioning [9, 18–20] with a relational, goal-orientated model of collaborative, therapeutic engagement that reduces parental alienation and stigma [16, 21]. The intervention delivered individually, offers parenting and self-care strategies and aims to develop a shared understanding between parent and clinician about how parents’ emotional and interpersonal difficulties impact on their parenting and the child’s functioning.

The qualitative research reported here was conducted in order to inform the design and methodology for a subsequent feasibility RCT of the Helping Families Programme [21]. The qualitative study sought to (i) examine the parenting and help-seeking experiences of parents affected by personality disorder, (ii) explore the acceptability of HFP to this population, and (iii) refine the protocol for the subsequent pilot RCT.

## Method

### Design

A qualitative design informed by Interpretative Phenomenological Analysis (IPA) [22, 23] was used to develop a rich understanding of the subjective lived experiences of participating parents and referring clinicians. IPA’s focus on generating meaning and significance from lived experience was important when exploring the acceptability of HFP. Ethical approval was obtained from the NRES Committee London (Camberwell St Giles).

### Sites & participants

Participants were recruited from four CAMHS teams in two London NHS trusts. Clinicians were asked to refer parents who were (i) affected by personality disorder, or likely to meet diagnostic criteria, and (ii) had a child (living with them) aged 3–11 years with a behavioural and/or emotional disorder. All referred parents and their referring clinician were eligible for qualitative interview. However, as described elsewhere [24] we screened for adult personality disorder and child mental disorders respectively, and excluded parents with a psychotic disorder, those in another psychoeducational parenting intervention, and those who’s child had a neurodevelopmental disorder or was on a child protection plan. Informed consent was obtained for all qualitative interviews.

### Participants

Five parents (all mothers, three lone parents) and their referring CAMHS clinicians (*n* = 5) participated in the qualitative interviews (i.e. 10 interviews). The sample size is consistent with IPA’s ideographic focus and the consensus in the literature on samples of this size [25]. *All parents either met research diagnoses or had clinical diagnoses of personality disorder (any) and their children met criteria for a behavioural and/or emotional problem. All children had siblings (range 1–2)*.

### Interviews

Separate semi-structured topic guides were developed for the parent and clinician interviews. In line with COREQ guidelines [26] we note the interviewer (RW) is a White-British female MA graduate, aged < 30 years, with no clinical responsibility for participants.

The researcher used the topic guides to build rapport and encourage open description of personal experiences [23] of parenting, help-seeking, their participation in HFP (if applicable) and related research processes [22]. Data were collected after participants had completed their participation in the case series. Interviews took place within the family home. Parents were given £10 to reimburse their time.

The referring clinicians topic guide explored their experience of working with the parents, and reflections on the parents involvement in the HFP. Clinicians were interviewed in their work place.

### Data analysis

Interviews were audio-recorded and verbatim transcripts obtained for analysis. Parent and clinician data were initially analysed separately and then triangulated to explore the relationship between their subjective experiences.

Data were analysed using the methods of IPA [22, 23] and coded at three levels: (i) the researcher familiarised herself with each transcript attaching descriptive codes containing initial observations and reflections to data, (ii) the researcher developed second-level descriptive labels which were then coded and organised into conceptual categories. Interpretations at this analytic stage were intended to capture the subjective value attributed by participants to emerging categories. For example, the theme ‘experience of parenthood’ incorporated the sub-codes containing emotional responses (e.g. frustration) and parenting behaviours (e.g. seeking support and negative experiences of parenting programmes). (iii) emergent results were subsequently explored and verified in shared meetings between authors (CD and TW) familiar with the transcripts. The codes, categories and themes emerging from the two respondent groups were examined and potential connections explored.

Results were subject to validation with a parent and a clinician participant. These latter procedures resulted in support for the emergent themes.

## Results

### Parent themes

### The experience of parenthood

Parents reported frequent problematic and distressing interactions with their children and the resulting negative impact on their family life. Parents described difficulties understanding and controlling their child’s behaviour.

*“...I’m hitting my head up against a brick wall because I just don’t know what else to do.” (Parent 3)*

The daily challenges of a child experiencing difficulties commonly coupled with a feeling of ‘helplessness’ about how to improve their situation appeared to define participants’ experiences of parenthood. Parents often appeared desperate and voiced a willingness to try whatever support was available, despite previous negative experiences of parenting interventions, variously described as ‘ineffective’, ‘inappropriate’ or ‘patronising’.

### Being a parent affected by personality disorder

Being a parent affected by personality disorder appeared to mediate many encounters with professionals. Some parents felt judged and blamed by clinicians. Parents felt professionals assumed that a personality disorder diagnosis automatically meant that they would be a *‘bad parent’*.

*“They were trying to say, ‘oh it’s down to your parenting, you were this, you were that .... it’s you that has given it to him’… I felt like they were blaming me.” (Parent 1)*

Consequently, parents felt clinicians did not take their parenting experiences seriously, perceived them to be a function of their interpersonal difficulties and therefore felt ‘unheard’ and unable to communicate their sense of helplessness.

Parents felt they had acquiesce to professional advice and intervention. Although often feeling pessimistic, they felt that they had to accept help offered on the clinicians terms as they would otherwise be seen as uncooperative.

### Experience of the intervention

Despite initial pessimism, after participation in HFP most parents described family life as ‘more manageable’ and their parenting challenges as ‘slightly easier’. Parents’ described successfully using various HFP parenting strategies (e.g. boundary setting, use of routines, spending time with the child), reported a greater awareness of the impact of their own parenting behaviour on their child, and expressed more interest and appreciation of their child’s own subjective experiences.

*“If I’m calmer and settled then obviously he’s gonna be a bit more calm and settled.” (Parent 1)*

As a result, parents felt a greater sense of agency in their parenting behaviour, more confidence and an increased sense of hope.

*“I can see that it has worked and see the changes.” (Parent 2)*

### Qualities of helping

Parents identified two key factors that encouraged their engagement in HFP. Parents attached value to perceived therapist personal qualities such as ‘encouraging’, ‘non-judgemental’, ‘open’, ‘honest’, ‘not patronising’ and ‘patient’. Parents felt listened to, understood and, as a result, encouraged and more in control. Illustrating this one parent described how session content was adjusted to reflect their personal circumstances and problems:

*“(If) my bipolar (lay description) was really bad or I felt really low and my depression was so bad, it wasn’t a matter of ‘right okay we’ve still got to do this’ ... (It was) let’s put this aside; let’s concentrate on what you need’ ” (Parent 3)*

These qualities were often described as absent in previous therapeutic interventions from which parents had disengaged.

### Clinicians

### Challenges addressing parental need

Clinicians described their challenges in meeting the needs of parents affected by personality disorder whose children had behavioural and emotional problems. Clinicians attributed some of these difficulties to systemic issues of funding, workload and ‘high thresholds’ in specialist services, and the organisational and cultural separation of CAMHS and adult services.

*“... they’re like two different worlds in what we provide.” (Clinician 2).*

Clinicians described the clinical challenges involved. Due to their priority focus on the child’s difficulties, at times, they felt that parents’ own psychological needs could be overlooked. Clinicians also felt less confident in assessing and managing parent’s mental health difficulties. Clinicians questioned the use of conventional parenting programmes for parents affected by personality disorder, voicing concerns about the group formats commonly used in parenting programmes and the potential for this to exacerbate parental emotional and interpersonal difficulties. Nevertheless, ongoing concerns about child and family functioning led clinicians to continue to offer support and intervention despite concerns about its limitations.

### Engaging parents in HFP

Clinicians expressed concern that personality disorder was a pejorative term that may have a detrimental impact on their therapeutic relationship. Citing previous cases when they had discussed personality and interpersonal functioning with parents and encountered defensiveness or disengagement, clinicians expressed reservations about discussing parental personality disorder in relation to HFP. In suggesting parent referral to another service, a clinician described how she had thought particularly carefully about her use of language to avoid the parent feeling blamed.

*“I’ve worked with her for quite a while and I was aware of the pattern that we’d been having in terms of we suggest a service, we get completely shut down ... there’s no way she’s actually gonna come round to this” (Clinician 1)*

### Limited involvement during HFP

Referring clinicians often had little contact with parents once they participated in HFP, due to the limited time they had to follow-up families who were not of immediate concern. Consequently, referring clinicians had limited knowledge of the impact of HFP on parents and their children. They were nevertheless positive about its potential value:

*“She’s (mother) not got in-touch with me. That’s a massive difference.” ... “ just really for this parent to have someone else to speak to and to understand her ... it’s definitely a benefit to her.” (Clinician 1)*

### Triangulation of parent and clinician themes

Parents had negative experiences with previous parenting interventions and clinicians were also aware of this. While parents were willing to participate, they and their referring clinicians felt pessimistic about the potential benefit of HFP prior to engagement.

Parents frequently felt their concerns about their child’s difficulties were unheard by previous clinicians. When trying to explain their family difficulties and sense of helplessness, parents felt stigmatised and blamed for their child’s problems because of their personality disorder diagnosis. This left parents feeling that their diagnosis ‘caused’ their children’s difficulties and undermined their efforts to obtain suitable help. At the same time, clinicians experienced difficulties in talking openly and effectively with parents affected by personality disorder because they held different views about the nature, cause and understanding of the family difficulties. Resolving these difficulties in ways that heighten parental engagement through a shared understanding was challenging for both parents and clinicians.

## Discussion

Our results provide insight into the experiences of parents affected by personality disorder, and the perspectives of the CAMHS clinicians with whom they work. The sample size, though small, is acceptable for a study using IPA. However, caution should be exercised in generalising the findings to other parents affected by personality disorder and other clinicians within and beyond CAMHS.

Analysis identified two negative parent themes related to (a) their sense of helplessness as parents and their resignation and pessimism about the effectiveness of parenting programmes, and (b) the feeling that clinicians in the past had erroneously attributed their family difficulties to their personality disorder. While parents felt desperate for change, based on past negative experiences, they were sceptical about achieving it through parenting interventions. Clinicians expressed similar doubts. Hence there was a shared ambivalence about the value of participation in the HFP intervention. This highlights the crucial role of sensitive and positive parental engagement and the value of developing tailored interventions designed to meet the needs of this population [10].

These findings are consistent with studies reporting stigma felt by parents with significant mental health difficulties [18]. While parenting difficulties may indeed be related to the symptomatic difficulties of the parent [5], in developing useful clinical formulations, other factors known to contribute to parenting difficulties need to be taken into account such as the child’s temperament and their own mental health difficulties [10, 27], lone parenthood [13] and social isolation [1, 27]. A comprehensive, ecological formulation that incorporates risk and resilience factors may provide a more accurate and acceptable basis for a shared understanding of child, parenting and family difficulties than a clinical approach that more narrowly focusses on parental mental health. While cautious interpretation is required, parents who did engage with HFP reported subjectively positive outcomes including changes to their parental behaviour, reflective function and emotional regulation.

### Clinical implications

Findings highlight potential barriers to engagement and participation for both parents and referring clinicians. To be successful, programmes such as HFP need to overcome this ambivalence and pessimism by engendering hope and motivation in parents and encouraging clinicians in their referral and gatekeeping roles [21]. Programmes also need to address the well documented engagement challenges for this population of parents, including underlying feelings of mistrust and difficulties in relating to others that are likely to interfere with building effective a therapeutic alliance [15].

Parents highlighted the subjective value of clinicians’ therapeutic consistency, flexibility, and relationships. These process characteristics may be more difficult to achieve within curricularised, group-based parenting programmes [5]. Offering tailored, individualised approaches, such as HFP, could give clinicians the opportunity to develop genuine open, shared understanding with parents about their difficulties without implying blame and judgement which become a barrier to treatment [21]. Additionally, a tailored, individualised approach would also enable the clinician to be flexible to differences in parenting styles across the spectrum of personality disorder diagnoses which, though beyond the scope of this paper, are well documented within the literature [7].

### Research implications

Targeted parenting interventions like HFP need to demonstrate their value to parents and clinicians through clinical outcome research. Though not methodologically robust in themselves, the current findings are consistent with and build upon previously published findings [11] and support the rationale for the pilot RCT currently in progress.

Our findings have informed recruitment strategies for the pilot RCT, which is recruiting parents with significant interpersonal difficulties rather than requiring parents have a formal research diagnosis of personality disorder. Recruitment will be through clinicians working within adult and child mental health services and social care practitioners. Participant and referring clinician information emphasises HFP’s use of a flexible, tailored approach based on individual, home-based delivery. Clinicians in referring teams have been encouraged to use the study’s findings to focus on, and showing genuine appreciation of, the difficulties parents face in caring for a child experiencing behavioural and emotional difficulties, rather than the ways in which the parents’ own interpersonal difficulties may make parenting challenging, or be the cause of and contribute to their child’s difficulties. Clinicians have been encouraged to be sensitive to the possible ambivalence that parents may feel and to openly explore the relative merits of involvement in HFP.

The results presented in this paper are based on small cohort of parents and clinicians which may limit generalisability. Parents supported by adult mental health services may have different perspectives on stigma and the role of diagnosis. The subsequent RCT has recruited parents through social care pathways. This will add further exploration and validation to these initial findings. Additional research methods including ethnography [28] and conversation analysis [29] may be useful to understand in greater depth the nature of the interactions between parents and clinicians particularly methods that can increase parent hope and motivation [20]. Also additional research looking at the trans-generational processes in which parental personality disorder impacts on child development through parenting practices [4, 7], including the role of child abuse and associated trauma, is required to further inform the intervention. Finally, the research focused on the experience of primary care-givers and help-seekers. The study did not examine the potential impact of a co-parent despite the sample including two-parent families. This is a limitation of the current study given wider evidence shows positive couple relationships are associated with lower levels of maternal stress and more positive parenting [21].

## Conclusions

This study provides new evidence from parents and referring clinicians about the experience of help seeking, parent-focussed support and participation in parenting interventions for parents with personality disorder. The results have implications for therapeutic engagement and intervention with this population of parents and their children who are at increased risk of poorer family outcomes and low engagement in parenting interventions. In response, changes have been made to HFP referral and recruitment procedures prior to conducting a pilot RCT of the intervention.

## Abbreviations

CAMHS: Child and adolescent mental health service
HFP: Helping Families Programme
IPA: Interpretative phenomenological analysis
RCT: Randomised control trial

## References

[1] Davis H, Day C, Cox A, Cutler L (2000). Child and adolescent mental health needs assessment and service implications in an inner city area. Clin Child Psychol Psychiatry.

[2] Fergusson D, Horwood L, Ridder E (2005). Show me the child at seven: the consequences of conduct problems in childhood for psychosocial functioning in adulthood. J Child Psychol Psychiatry.

[3] Loeber R, Farrington D (2000). Young children who commit crime: epidemiology, developmental origins, risk factors, early interventions, and policy implications. Dev Psychopathol.

[4] Dutton DG, Denny-Keys MK, Sells JR (2011). Parental personality disorder and its effects on children: a review of current literature. J Child Custody.

[5] Stepp SD, Whalen DJ, Pilkonis PA, Hipwell AE, Levine MD (2011). Children of mothers with borderline personality disorder: identifying parenting behaviors as potential targets for intervention. Personal Disord.

[6] Newman LK, Stevenson CS, Bergman LR, Boyce P (2007). Borderline personality disorder, mother-infant interaction and parenting perceptions: preliminary findings. Aust N Z J Psychiatry.

[7] Johnson JG, Cohen P, Kasen S, Ehrensaft MK, Crawford TN (2006). Associations of parental personality disorders and axis I disorders with childrearing behavior. Psychiatry.

[8] Early Intervention Foundation Foundations for Life (2016). What works to support parent child interaction in the early years.

[9] National Institute for Health and Clinical Excellence (NICE) (2013). Antisocial behaviour and conduct disorders in children and young people: recognition and management (Nice guideline CG158).

[10] Reyno SM, McGrath PJ (2006). Predictors of parent training efficacy for child externalizing behavior problems: a meta-analytic review. J Child Psychol Psychiatry.

[11] Maliken AC, Fainsilber KL (2013). Exploring the impact of parental psychopathology and emotion regulation on evidence-based parenting interventions: a transdiagnostic approach to improving treatment effectiveness. Clin Child Fam Psychol Rev.

[12] Kazdin AE, Wassell G (1999). Barriers to treatment participation and therapeutic change among children referred for conduct disorder. J Clin Child Psychol.

[13] Armstrong H (2007). Ministerial foreword. Reaching out: think family.

[14] Bee P, Bower P, Byford S, Churchill R, Calam R, Stallard P, et al. The clinical effectiveness, cost-effectiveness and acceptability of community-based interventions aimed at improving or maintaining quality of life in children of parents with serious mental illness: a systematic review. NIHR J Library. 2014; 10.3310/hta18080. 10.3310/hta18080 PMC4780907 24502767

[15] McMurran M, Huband N, Overton E (2010). Non-completion of personality disorder treatments: a systematic review of correlates, consequences, and interventions. Clin Psychol Rev.

[16] Day C, Ellis M, Harris L (2012). Helping families programme manual.

[17] Day C, Kowalenko S, Ellis M, Dawe S, Harnet P, Scott S (2011). The helping families programme: a new parenting intervention for children with severe and persistent conduct problems. Child Adolesc Mental Health.

[18] Ackerson B (2003). Coping with the dual demands of severe mental illness and parenting: the parents’ perspective. Fam Soc.

[19] Barlow J, Jarrett P, Mockford C, McIntosh E, Davis H, Stewart-Brown S (2007). Role of home visiting in improving parenting and health in families at risk of abuse and neglect: results of a multicentre randomised controlled trial and economic evaluation. Arch Dis Child.

[20] Newton-Howes G, Weaver T, Tyrer P (2008). Attitudes of staff towards patients with personality disorder in community mental health teams. Aust N Z J Psychiatry.

[21] Davis H, Day C (2010). Working in partnership with parents.

[22] Smith J, Osborne M, Smith J (2003). Interpretive phenomenological analysis. Qualitative psychology: a practical guide to research methods.

[23] Smith JA (1995). Semi-structured interviewing and qualitative analysis. Rethinking Methods Psychol.

[24] Day C, Briskman J, Crawford M, Harris L, McCrone P, McMurran M (2017). Feasibility trial of a psychoeducational intervention for parents with personality difficulties: the helping families programme. Contemp Clin Trials Commun.

[25] Brocki JM, Wearden AJ (2006). A critical evaluation of the use of interpretative phenomenological analysis (IPA) in health psychology. Psychol Health.

[26] Tong A, Sainsbury P, Craig J (2007). Consolidated criteria for reporting qualitative research (COREQ): a 32-item checklist for interviews and focus groups. Int J Qual Health Care.

[27] Feinstein EBl. What works to enhance inter-parental relationships and improve outcomes for children. Early Intervention Foundation. 2016. http://dera.ioe.ac.uk/25869/1/what-works-to-enhance-inter-parental-relationships.pdf. Accessed 18 Sept 2017.

[28] Hammersley M (2007). Atkinson P.

[29] Hutchby I, Wooffitt R (2008). Conversation analysis.

